# A Review of Liposomes as a Drug Delivery System: Current Status of Approved Products, Regulatory Environments, and Future Perspectives

**DOI:** 10.3390/molecules27041372

**Published:** 2022-02-17

**Authors:** Peng Liu, Guiliang Chen, Jingchen Zhang

**Affiliations:** Shanghai Center for Drug Evaluation and Inspection, Haiqu Road 58, Shanghai 201210, China; chenguiliang@smda.sh.cn

**Keywords:** liposomes, drug delivery, lipid excipient, drug loading, marketed products

## Abstract

Liposomes have been considered promising and versatile drug vesicles. Compared with traditional drug delivery systems, liposomes exhibit better properties, including site-targeting, sustained or controlled release, protection of drugs from degradation and clearance, superior therapeutic effects, and lower toxic side effects. Given these merits, several liposomal drug products have been successfully approved and used in clinics over the last couple of decades. In this review, the liposomal drug products approved by the U.S. Food and Drug Administration (FDA) and European Medicines Agency (EMA) are discussed. Based on the published approval package in the FDA and European public assessment report (EPAR) in EMA, the critical chemistry information and mature pharmaceutical technologies applied in the marketed liposomal products, including the lipid excipient, manufacturing methods, nanosizing technique, drug loading methods, as well as critical quality attributions (CQAs) of products, are introduced. Additionally, the current regulatory guidance and future perspectives related to liposomal products are summarized. This knowledge can be used for research and development of the liposomal drug candidates under various pipelines, including the laboratory bench, pilot plant, and commercial manufacturing.

## 1. Introduction

Liposomes are self-assembled (phospho)lipid-based drug vesicles that form a bilayer (uni-lamellar) and/or a concentric series of multiple bilayers (multilamellar) enclosing a central aqueous compartment [1]. The size of liposomes ranges from 30 nm to the micrometer scale, with the phospholipidbilayer being 4–5 nm thick [2]. The field of liposomology was launched by the British scientist Alec Bangham and colleagues at Babraham Cambridge in the mid-1960s [3], and they first published the structure of liposomesin 1964 [4]. Since then, liposomes have been widely investigated as delivery vehicles for small molecular drugs, protein, nucleic acid, and imaging agents [5,6,7,8,9]. Different administration routes, such as parenteral, pulmonary, oral, transdermal, ophthalmic, and nasal routes, have been developed to improve therapeutic efficacy and patient compliance [10,11,12,13,14]. In addition, liposomes have been widely applied in the fields of food [15] and cosmetics [16].

As drug vehicles, liposomes exhibit outstanding properties, such as protecting the encapsulated substances from physiological degradation [17], extending the half-life of the drug, controlling the release of drug molecule s [18], and excellent biocompatibility and safety. Furthermore, liposomes can selectively deliver their payload to the diseased site through passive and/or active targeting, thus decreasing the systemic side-effect, elevating the maximum-tolerated dose, and improving therapeutic benefits [19,20].

Unlike normal tissue with tight intracellular junctions (2–6 nm) between endothelial cells [21], abnormal tissues such as a solid tumor or inflammatory site have highly porous capillaries (100 nm–2 µm depending upon the size and type of tumor tissue [22]). Liposomes can cross over the discontinuous neovasculature and be passively accumulated and detained at the abnormal tissues, which is called the enhanced permeability and retention (EPR) effect. Actively targeting employs specific interactions between the ligands and receptors on the surface of liposomes and tumor cells, respectively. Tumor cells may overexpress specific receptors, such as vascular endothelial growth factor (VEGF), epidermal growth factor (EGF), folic acid (FA), integrin, CD44 (a cell surface glycoprotein), CD13, and prostate-specific membrane antigen [23]. According to these receptors, specific ligands, such as antibody [24], nuclear acid (e.g., aptamers [25]), protein (e.g., transferrin [26]), peptides (e.g., iRGD [27], iNGR [28]), small molecules (folic acid [29]), and carbohydrates (e.g., dextran, mannose, and galactose [30], targeting macrophages) were proposed for the surface modification of liposomes.

Besides the specific medicines, liposomes stand as an excellent technique for drug delivery. However, only 14 types of liposomal products are available on the market, which means the advantages of liposomes have not been fully exploited. Therefore, in this review, we summarized the knowledge about commercial liposomal products approved by the FDA and EMA. Attention is paid to the composition and manufacturing technologies adopted in commercial products. In addition, the CQAs of liposomes, the current regulatory environment, and future perspectives are introduced. The purpose of this review is to provide important reference information to accelerate the development of liposomes.

## 2. The Marketed Liposomal Products

We searched the approved drug database published on the website of the FDA and EMA, and found that 14 types of liposomal products have been authorized (Table 1). It should be noted that this list excludes generics, lipid complexes (e.g., Abelcet, Amphotec, and Onpattro), and nationally authorized liposomal products in Europe. Doxil(Doxorubicin HCl liposome injection) was the first liposomal product approved by the FDA in 1995. Among these marketed products, 43% of products were approved before the year 2000, and 57% of products were approved before the year 2010. The therapeutic area mainly focuses on cancer therapy but also involves other areas, such as infection, anesthesia, vaccine, lung disease, and photodynamic therapy. The dosage forms are mainly focused on sterile suspension and lyophilization powder. The administration routes include intravenous infusion, intramuscular and intrathecal injection, epidural, local infiltration, and oral inhalation.

## 3. Structures and Main Components of Liposomes

### 3.1. Structures of Liposomes

**Figure 1:**
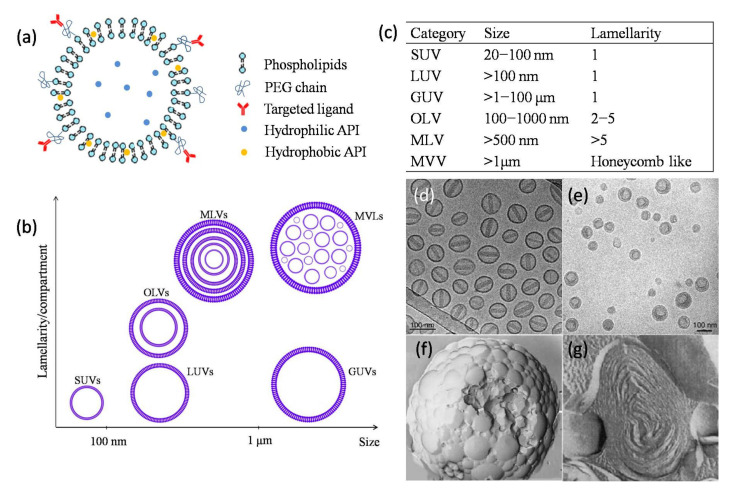
Categories and structures of liposomal drug delivery system. (**a**) Structural illustration of liposome composition. The size of a typical phospholipid bilayer is 4.5 nm, which is much smaller than the one of the inner aqueous core; (**b**) Classification of liposomal vesicles according to their lamellarity/compartment and particle size; (**c**) The size and lamellarity of different types of liposomes; (**d**,**e**) The cryo-transmission electron microscopy of Doxil [35] and Vyxeos [36]; (**f**,**g**) The electron micrographs of DepoFoam^TM^ particles with a typical diameter of 1–100 μm (e.g., DepoCyt) and MLVs with a typical diameter of 0.2–5 μm (e.g., Mepact) [37].

Liposomes can be classified as unilamellar vesicles (ULVs), oligolamellar vesicles (OLVs), multilamellarvesicles (MLVs), and multivesicular liposomes (MVLs) depending on the compartment structure and lamellarity (Figure 1) [31]. OLVs and MLVs show anonion-like structure but present 2–5 and >5 concentric lipid bilayers, respectively. Different from MLVs, MVLs include hundreds of non-concentric aqueous chambers bounded by a single bilayer lipid membrane and display a honeycomb-like structure [32]. Based on the particle size, ULVs can be further divided into small unilamellar vesicles (SUVs, 30–100 nm), large unilamellar vesicles (LUVs, >100 nm), and giant unilamellar vesicles (GUVs, >1000 nm) [33]. Different size range of ULVs was reported, i.e., SUVs with a size of less than 200 nm and LUVs with a size of 200–500 nm [34].

The particle size and the structures of the commercial products are concluded in Table 2. Most of the current commercial products are SUVs (e.g., Doxil (Figure 1d) because of the long circulation time and ability to passively target the diseased site. Arikaye (amikacin liposome inhalation suspension) is considered an LUV because of its large particle size (200–300 nm). Vyxeos (daunorubicin: cytarabine liposome for injection) is a bilamellar liposome system (Figure 1e), which is generated during the first drug cytarabine loading procedure. The mechanism of internal lamella formation is explained as a thermodynamic response of the lipid bilayer to decrease the surface area-to-volume ratio of the liposomes caused by water egress in response to an external osmotic challenge [36]. Myocet (liposomal doxorubicin) and Mepact (liposomal mifamurtide powder for concentrate for dispersion for infusion) (Figure 1g) are MLVs. The abundant lamellae provide a large space for the encapsulation of lipophilic compounds [38].

There are four products with micron diameters, i.e., Mepact, DepoCyt (cytarabine liposome suspension), DepoDur (morphine sulfate extended-release liposome injection), and Exparel (bupivacaine liposome injectable suspension). Mepactis a sterile and lyophilized cake, and will form multilamellar liposomes with a particle size of 2.0–3.5 µm after reconstituted with 0.9% saline solution. This size is ideal for recognition and phagocytosis by monocytes/mocrophages, and triggers the macrophages and the immunomodulatory effects for cancer therapy. DepoCyt, DepoDur, and Exparel are manufactured by the same DepoFoam technique. The MVLs structure (Figure 1f) can load a large volume of drug–aqueous solution because of the numerous chambers and provide sustained release due to the erosion/degradation of liposomes and diffusion of drug molecules [39,40].

### 3.2. Main Components of Liposomes

**Figure 2:**
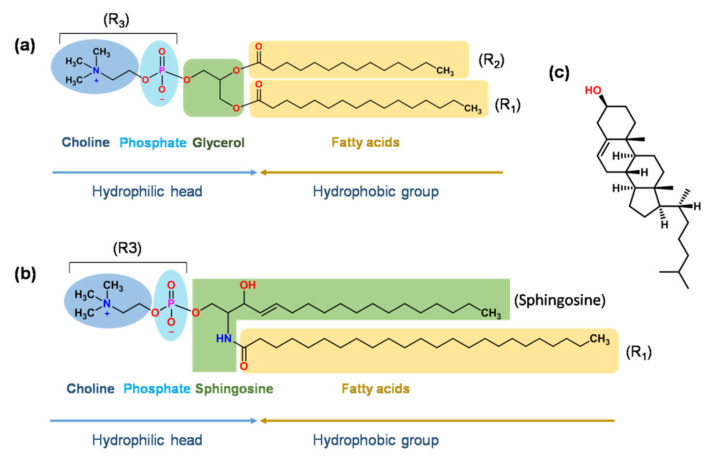
(**a**) Structural illustration of glycerolphospholipid. R1 and R2 can be saturated or unsaturated fatty acids, such as decanoic acid, lauric acid, palmitic acid, oleic acid, myristic acid, stearic acid, and erucic acid. R3 can be phosphatidylcholine (PC), phosphatidyl ethanolamine (PE), phosphatidyl serine (PS), phosphatidyl inositols (PI), phosphatidic acid (PA), phosphatidylglycerol (PG), and cardiolipin; (**b**) Structure of sphingomyelin. (**c**) Structure of cholesterol.

Table 2 shows that glycerolphospholipid (GP), sphingomyelin (SM), and cholesterol (Chol) are the basic components used in the marketed products. GP contains glycerol, which links a pair of hydrophobic fatty acid chains and a hydrophilic polar head group [48]. The types of fatty acid and polar heads are described in Figure 2a. Under the physiological pH, different head groups provide liposomes with negative (PA, PS, PG, and cardiolipin) or neutral (PC and PE) charges [49].

Negatively charged DSPG used in AmBisome (ambisome liposome for injection) can interact with the positively charged amine group of AmpB to form a stable ionic complex [50], while DSPG used in Vyxeos minimize liposome aggregation by a strong Coulombic repulsive force [51]. DSPC used in DaunoXome (daunorubicin citrate liposome injection), Onivyde (irinotecan liposome injection), and Vyxeos is a neutral and synthetic lipid with well-defined fatty acid composition (two molecules of stearic acid), high purity, and a relatively high phase transition (T_m_ of 55 °C).

EPC is adopted as an excipient in Myocet and Visudyne (verteporfin powder for solution for infusion). EPC is a naturally sourced phospholipid (NPL) purified from egg yolk. Compared to semi-synthetic and synthetic lipids, NPL exhibit a low production cost, but a broad transition temperature, problematic to obtain completely identical NPL and potential batch variation of liposomes [52]. In addition, the unsaturated fatty acid of EPC leads to a low phase transition temperature of −15~−5 °C [53], indicating the liposome bilayer is in a disorder and drug “leaky” state in the body temperature. Myocet, composed of EPC, is unstable in blood, and most drugs are released after 24 h [54]. Visudyne composed by EPC and DMPC also exhibits less stability in the presence of serum. The verteporfin rapidly transfers from the disordered liposome membrane and associates with the plasma lipoproteins, then reaches higher levels in the neovasculature since low-density lipoprotein (LDL) receptors are abundant in neovascular tissue [55,56].

MethoxyPEG (Mw 2000 Da), covalently attached to DSPE (MPEG-DSPE) used in Doxil and Onivyde, provides “stealth” and sterically stabilized liposomes. The molecular weight of PEG and the mole percentage of PEG-DSPE in lipid composition play important roles on the bilayer packing, circulation time, and thermodynamic stability. The high molecular weight of PEG (>2000 Da) grafted to the lipid headgroup exhibits repulsive forces from the liposome surface, as well as protects liposomes from binding serum proteins and avoids further clearance by the mononuclear phagocytic system (MPS), but also decreases the interaction and endocytosis of liposomes by targeted cells [57]. The low molecular weight of PEG (<750 Da) shows an insignificant steric stabilization effect [58]. Additionally, the highest biological stability of liposomes can be obtained when the concentration of PEG-DSPE is 7 ± 2 mol% in the lipid assemblies, and 5 mol% of PEG-lipid conjugates as a typical concentration have been used in vivo (e.g., Doxil) [58,59]. In the case of the concentration of PEG-DSPE below 4 mol%, the PEG chains shows “mushroom” configuration with a thickness about 3.5 nm. As the increased concentration of 4–8 mol%, the configuration of PEG chains transforms to “brush” with a thickness of 4.5–10 nm [58,60]. Further increasing the molar ratio, micelles are formed instead of liposome assembling.

DepoCyte, DepoDur, and Exparel have special structures and similar lipid components. A minimum of two types of lipids in the formulation are required for MVLs formation, the amphipathic lipid and the neutral lipid (e.g., diglycerides, triglycerides, vegetable oil) [61]. DOPC and DEPC are amphipathic zwitterionic phospholipids that form the walls of honeycomb-like chambers. DPPG with a negative charge prevents the MVLs from aggregation [62]. Neutral lipids (e.g., triolein and triglycerides) act as a hydrophobic space filler at bilayer intersection points and stabilize these membrane junctions [63]. Without the neutral lipids, conventional ULVs or MLVs will be formed instead of MVLs. The amount of neutral lipids used in formulation decides the capture volume and encapsulation efficiency of MVLs [63].

GPs play a key role in formulation since they affect the biophysical properties of liposomes (e.g., drug encapsulation, stability, and drug release) and further influence the pharmacokinetic behavior and pharmacodynamics in vivo [64]. The length, symmetry, inter- and intra-molecular interactions, branching, and unsaturation degree of hydrocarbon chains decide the thickness and fluidity of the bilayer, phase transition temperature, and drug release rate [49,65]. In brief, the longer hydrocarbon chain could induce a tighter membrane packing and increase drug retention, whereas the higher degree of unsaturation or branching of the hydrocarbon chain could result in looser membrane packaging, which is probably caused by the preferential interaction of cholesterol with saturated phospholipids in comparison with unsaturated ones [66,67].

Sphingomyelin (SM) (Figure 2b) has a similar structure toa glycerolphospholipid, except that glycerol is replaced by sphingosine [68]. Marqibo (vincristine sulfate liposome injection) uses SM to form the bilayer membrane, which significantly decreases the lipid hydrolysis in an acidic environment and prompts the stability of liposomes. An acid environment (pH 2.0–4.0) is routinely used for producing a transmembrane pH gradient for active drug loading. Under the condition of 37 °C and pH 2.0, the rate of hydrolysis for liposomes was approximately 100-fold slower in SM/Chol (55/45, mol/mol) liposomes than in DSPC/Chol liposomes [69]. In addition, liposomes with SM/Chol showed optimal pharmacokinetic properties, i.e., increased circulation time and enhanced delivery of the drug to target tissues [70].

Cholesterol (Chol) (Figure 2c) is another main component of the liposome bilayer and can be used in almost all commercial products (Table 2). The addition of Chol can promote the packing of lipid chains and bilayer formation [71], modulate the fluidity/rigidity of membrane [72], and further affect the drug release [73,74], stability of liposomes [75], and the kinetics of exocytosis [76]. For the product of Shingrix (herpes zoster vaccine, contains glycoprotein E antigen and AS01_B_ liposomal adjuvant system), Chol can avoid the hydrolysis of QS21 (one of the immunoenhancers in the AS01_B_ adjuvant system) at a ratio of 2:1 (Chol: QS21, *w*/*w*) [77]. For the product of AmBisome, Chol reduces the toxicity of the liposomal formulation compared with the non-sterol one [41]. The effect of Chol on the bilayer’s properties is concentration-dependent. It was reported that [71] the low (2.5 mol%) and high (>30 mol%) concentrations of Chol showed little effect on the properties of the lipid bilayer. The “condensing effect” or “ordering effect” of Chol with the content of 5 < Chol mol% < 30 led to a gradual increase in particle size from 220 nm to 472 nm, a decrease in the fluidity of membrane, and a decrease in the release of drug. Besides Chol, other sterols, such as progesterone, ergosterol, and lanosterol, with a similar structure to Chol were also investigated to modulate the membrane rigidity and stability [74,78].

## 4. Manufacturing Process

**Figure 3:**
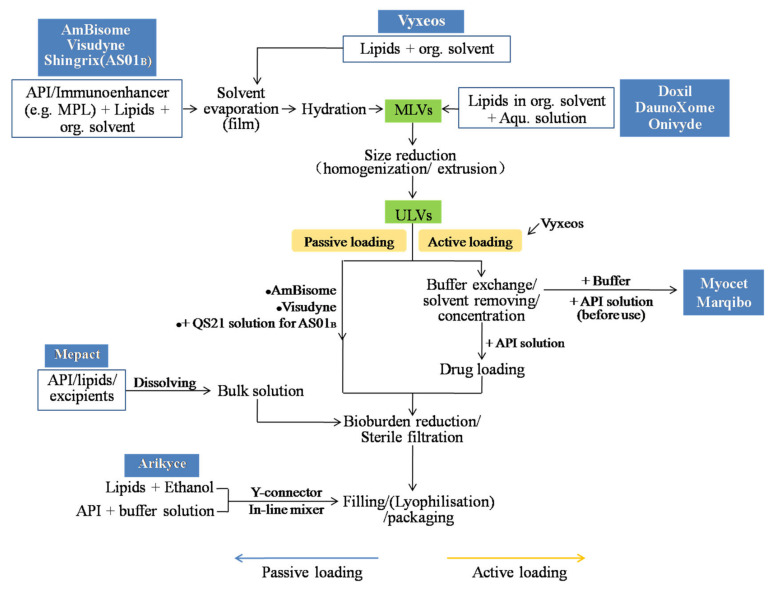
The potential manufacturing processes of the marketed liposomal products, summarized based on the related patents or publications.

Various preparation methods of liposomes have been developed. The potential manufacturing processes of the marketed liposomal products summarized based on the related patents and publications are shown in Figure 3 [79]. The commonly used manufacturing processes include the thin-film hydration, ethanol injection, and double emulsion methods. The processes routinely include (1) the preparation of MLVs or ULVs depending on the choice of methods; (2) size reduction if necessary; (3) preparation of the drug solution(s) and drug loading, while this step is combined with step 1 in the case of passive drug loading; (4) buffer exchange and concentration if necessary; (5) sterile filtration or aseptic processing; (6) lyophilization, if needed, and packaging.

### 4.1. Liposome Preparation

#### 4.1.1. Film-Hydration Method

The thin-film hydration method is a traditional technique and is beneficial for loading the lipophilic drug. A thin film is created by evaporating the lipid–solvent solution during flask rotation under vacuum. MLVs suspension can be obtained by adding the aqueous solution to hydrate the lipid film. The particle size can be further reduced to obtain SUVs, and the drug substance can be passively or actively loaded during or after the liposome formation, respectively. The commercial products of AmBisome, Visudyne, and Shingrix (Adjuvant systemAS01_B_) adopt this method for manufacturing [56,77,80]. For example, Visudyne is manufactured through evaporating the ingredients from dichloromethane, hydration with lactose solution, size reduction by homogenization, filtration, and lyophilization. Adjuvant systemAS01_B_ is an individual vial in the product of Shingrix, and is a liposome-based adjuvant containing two immunoenhancers, QS21 (a triterpene glycoside purified from the bark of the tree *Quillajasaponaria* Molina) and MPL (3-Odesacyl-4′-monophosphoryl lipid A). The MPL and other lipids are dissolved in the organic solution and dried. After hydration and size reduction, the QS21 aqueous solution is added for formulation.

#### 4.1.2. Double-Emulsification Method

This technique, also known as DepoFoamplatform^TM^, has been adopted by three commercial products of DepoCyte, DepoDur, and Expel to produce MVLs. The whole production routinely includes four sequential operations as follows: (1) the formation of a “water-in-oil” emulsion, (2) the formation of a “water-in-oil-in-water” emulsion, (3) solvent extraction with the help of stripping gas or vacuum pressure, and (4) microfiltration for the removal of the free drug, concentration, and exchange of external solution [37,63]. During the manufacturing process, aseptic assurance should be provided since MVLs owing the micro particle size cannot be produced as sterile batches through the 0.22 µm filtration. Lu et al. [81] studied the influence of the process on critical quality attributes of bupivacaine MVLs and found that the particle size of the first emulsion increases with the increase in lipid concentration, and shearing speed strongly influences the particle size. For the second emulsion, the encapsulation efficiency decreases during the solvent removal since some MVLs are collapsed and the drug leaks from the internal aqueous phase. Additionally, the high temperature promotes the mobility and rearrangement of lipid bilayers, resulting in lipid fusion and the collapse of the aqueous chambers.

#### 4.1.3. Solvent Injection Techniques

For this kind of technique, lipid materials and lipophilic substances are dissolved in a water-miscible organic solvent, and then the organic phase is injected into a large amount of aqueous buffer, resulting in small unilamellar liposomes being spontaneously formed [82]. In other modified methods, two streams of solution are injected/infused through the Y-connector [83] and membrane contactors in a tubular (e.g., Shirasu Porous Glass membrane [84] or hollow fiber configuration [85]) and crossflow injection [86,87] device to improve the micromixing of the organic phase into the aqueous phase. The solvent rapidly diffuses in an aqueous medium, and interfacial turbulence leads to the formation of small and homogenous liposomes [88]. The particle size between 80 nm and 300 nm can be prepared depending on the preparation conditions [89], and the additional energy input for particle size reduction, such as sonication and extrusion, is not required. The organic solvent should be removed using evaporation, lyophilization, dialysis, or diafiltration, and the liposomes suspensions can be concentrated to the desired volume. Ethanol is commonly used as an organic solvent because of its safety. Various preparation parameters, including the flow rate, the temperature of both solvent and aqueous solution, the lipid concentration, as well as the stirring rate, can affect the properties of particles [88]. Arikayce uses “ethanol infusion” or “in-line infusion” to prepare amikacin liposomes [64]. The minimal amount of lipids–ethanol solution and the amikacin sulfate aqueous solution are mixed by a Y-connector and in-line mixer to form the nanosized amikacin liposomes.

#### 4.1.4. In Situ Preparation of Liposomes

“In situ” is regarded as liposomes that are formed before clinical use [90]. The commercial product of Mepacthas adopted this method for production. Drug and phospholipids are formulated into a bulk solution, and filtration for sterilization, filling, and lyophilization is followed. In the case of Mepact, only three components, i.e., active ingredient muramyl tripeptide phosphatidyl ethanolamine (MTP-PE), palmitoyl-oleoyl-phosphatidylcholine (POPC) and dioleoyl-phosphatidylserine (OOPS) with a certain ratio (POPC:OOPS = 7:3, MTP-PE:phospholipids = 1:250) are included [91]. The product is a dry lipid cake with a porous structure, providing a large surface area for contact with the constitution medium. Before clinical use, 0.9% saline solution is added into the vial, and the dry substance is hydrated to form multilamellar liposomes with a particle size of 2.0–3.5 µm [92] and monomodal size distribution. The phase transition temperature of phospholipids in water is about 5 °C, which allows liposome preparation in situ at room temperature [93].

### 4.2. Size-Reduction Techniques

Size and size distribution are critical attributions for the performance and safety of liposomes. Several methods are available for the size reduction of liposomes, such as (ultra)sonication either by bath or probe, French press [94], extrusion, homogenization, or combination methods, such as freeze–thaw extrusion [95], freeze–thaw sonication, and a high-pressure homogenization–extrusion technique [96]. Among these techniques, extrusion and high-pressure homogenization (HPH) are the most popular used in pharmaceutical manufacturing.

The extrusion technique was first introduced in 1971 [97]. Liposomes of large sizes pass through the polycarbonate membranes (50 nm~5 µm) or asymmetric ceramic filter to become the smaller one with a fine size distribution. It is known that commercial nano-liposomal products, including Onivyde, Vyxeos, and Marqibo, use this method for production. This method is relatively simple, reproducible, and only moderate conditions are required. The potential mechanism of size reduction is that MLVs are ruptured at the entrance of the membrane pore and rearranged during the membrane passage [98,99]. The critical process parameters, such as the pore size of the polycarbonate membrane, the number of passage cycles, pressure, and flow rate, can influence the size and liposomal lamellarity [100]. Ong et al. [101] found that extrusion was the most efficient technique when comparing other different nanosizing techniques, including freeze–thaw sonication, (ultra)sonication, and homogenization. However, extrusion may decrease the liposome encapsulation and change the structure of asymmetric liposomes [102].

HPH is employed to produce various nano-formulations, such as liposomes, nanocrystals, and nanoemulsions. It is suitable for both aqueous and non-aqueous systems and provides different production scales, from the laboratory scale with 10 L/h capacity to large production scales with 100,000 L/h capacity [103]. Commercial liposomal products, including Visudyne and AmBisome, use this method for manufacturing. The MLVs suspension is passed through a narrow gap under high pressure, broken down by means of shear force, turbulence, and cavitation of fluid generated by the velocity gradient, and then rearranged into smaller liposomes. The particle size and size distribution are decided by the parameters of the homogenization process, such as pressure, processed cycles, valve and impingement design, and flow rate; they are also affected by the properties of samples, including the composition and viscosity of the bulk medium and initial size distribution of particles. The increasing pressure and processed cycles decrease the particle size and polydispersity index (PDI), but also resulting in a decrease in the encapsulation efficiency [104,105].

### 4.3. Drug-Loading Methods

High drug loading is desirable to minimize the amount of excipient, reach the desired concentration of therapeutic agents, decrease the dose volume, and reduce dosing time. Two primary techniques are routinely used for drug loading, i.e., passive and active drug loading procedures. Additionally, there are some other drug-entrapment methods, such as a drug–lipid chemical conjugate, the combination of passive and active drug encapsulation.

#### 4.3.1. Passive Drug-Loading Approach

The passive drug-loading method involves encapsulating the drug agent during the preparation of liposomes. The drug can be encapsulated within the inner aqueous space or embedded in the bilayer of liposomes by means of covalent, ionic, electrostatic, non-covalent, or steric interactions between drug molecules and lipids. The main disadvantage of this approach is the low encapsulating efficiency, and thus leading to an additional step of free drug removal. From the learn of patents and publications, the marketed liposomal products using the passive drug loading method include AmBisome, Visudyne, Arikayce, DepoCyte, DepoDur, and Expel.

##### For Lipophilic Drug Substance

Verteporfin, also known as Benzporphyrin Derivative Monoacid Ring A (BPD) (Visudyne), is a highly lipophilic molecule, which can promote drug participation efficiently into the lipid bilayer. The entrapment efficiency of BPD in liposomes is almost 100% after homogenization [43].

AmpB (AmBisome) is poorly soluble in aqueous and in most organic solvents because of its amphipathic structure. AmpB can be tightly intercalated into the lipid bilayer by the ionic association between the positively charged amino group of AmpB and the negatively charged phosphate group of DSPG. The ionic interaction is easily formed in an acid environment of pH 1.0–3.0 [106]. In addition, the association is further strengthened by the hydrophobic interactions between the polyene portion of AmpB and aliphatic hydrocarbon chains of phospholipids.

##### For a Hydrophilic Drug Substance

Amikacin sulfate (Arikayce) is a freely water-soluble, anti-infective drug. Due to the limited solubility of amikacin in ethanol, amikacin transfers to a semi-soluble, coacervated state entrapped inside the core of liposomes during the liposome preparation using ethanol infusion [107]. Surprisingly, high encapsulation efficiencies (free drug 5.2% with the optimized preparation parameters) and drug-to-lipid ratio (~0.7) were obtained [83]. The encapsulated drug exhibits a low permeability from the liposome membrane because of its multi-cationic nature, rendering stable liposomes during the circulation in blood [108].

Cytarabine (DepoCyte), Morphine (DepoDur), and Bupivacaine (Exparel) aqueous solution encapsulated in the chambers of MVLs, which consists of 94% aqueous chambers and 4% lipids) [109]; therefore, a small volume of liposome suspensions contains large quantities of drugs. In order to further improve the encapsulation efficiency and sustained release, the conversion of drug compounds from monoprotic mineral acid salts into diprotic or triprotic mineral acid salts (e.g., sulfate salt or phosphoric salt) and co-encapsulating of polyalcoholic organic acids can be used [110].

#### 4.3.2. Active Drug-Loading Approach

The active drug loading approach, also called remote drug loading, involves loading the drug agent after empty liposomes are produced. The transmembrane gradient of pH or ion concentration is the driving force to promote the drug diffuse across the membrane into the inner core of liposomes. The drug-entrapment process takes around 5 min to 30 min, and a high loading efficiency (above 90%) can be reached.

**Figure 4:**
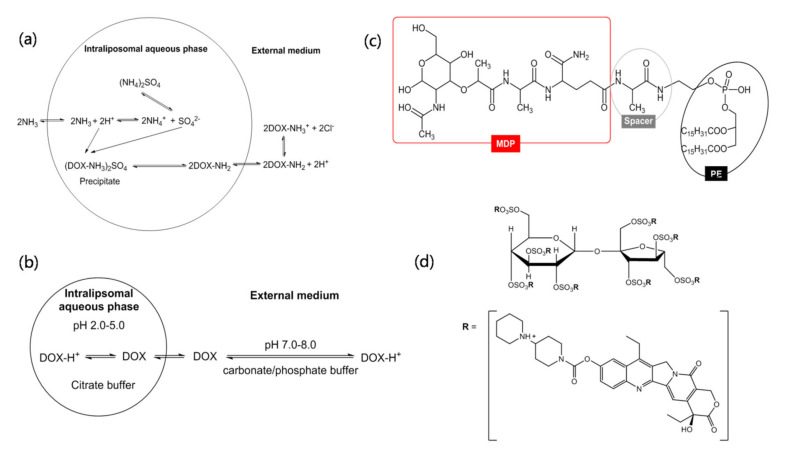
Different mechanisms of remote drug loading. (**a**) Doxil: DOX-loaded by transmembrane gradient of (NH_4_)_2_SO_4_ concentration [35]; (**b**) Myocet, Marqibo, and DaunoXome: drug loaded by transmembrane gradient of pH; (**c**) Mepact: MDP chemically conjugated to PE through a peptide spacer, then formed liposomes with other phospholipids. (**d**) Onivyde: irinote can loaded by transmembrane gradient of the concentration of sucrosofate triethylammonium salt (TEA-SOS). One molecule of SOS can bind 8 molecules of irinotecan.

Doxil is a typical example of drug loading based on the transmembrane gradient of ammonium sulfate (Figure 4a). Due to the concentration of (NH_4_)_2_SO_4_in the core of liposomes being far higher than the external medium, the neutral molecules of DOX-NH_2_ with high permeability and Octanol-to-buffer partition coefficients diffuse across the lipid bilayer and enter the liposome’s inner aqueous phase. The (DOX-NH_3_)_2_SO_4_ precipitation with a fiber-like crystalline form is generated in the core of the liposome. The low solubility of (DOX-NH_3_)_2_SO_4_ minimizes the intraliposomal osmotic pressure and thus keeps the liposome integrity.

For the product of Myocet, DOX is loaded before clinical use. Transmembrane pH gradient is the driving force for DOX loading (Figure 4b). Myocet has three vials in one package, including vial 1—doxorubicin HCl red lyophilized powder, vial 2—liposomes suspensions in 300 mM citric acid at pH 4–5, and vial 3—sodium carbonate buffer [46]. Before clinical use, empty liposomes (vial 2) are injected into the sodium carbonate buffer (vial 3) to adjust the exterliposome medium to a pH of 7–8, and then are mixed with DOX saline solution. The neutral form of DOX molecules (pKa = 8.3) in the exterliposome medium cross the liposomal bilayer and form a unique DOX-citrate complex in the vesicle interior. The DOX-citrate complex exhibits bundles of flexible fiber, attributing to DOX monomers owning a relatively flat ring stack together to form fibers [111]. The loading efficiency is above 95%. Similar to Myocet, Marqibo also has three vials in one package. The empty liposomes have the inner aqueous phase of citrate buffer (0.3 M, pH about 4.0) [112]. Before vincristine sulfate (pKa = 5.4) loading, the external pH of liposomes is increased to about pH 7.0–7.5 by adding sodium phosphate buffer at a concentration of 14.2 mg/mL.

Different from Myocet and Marqibo, DaunoXome employs a low pH gradient (citric acid, 50 mM), resulting in a relatively weaker daunorubicin loading and then a short half-life of the drug and low AUC [106]. Oppositely, a high transmembrane pH gradient (e.g., intraliposomal pH 2.0) can increase the drug encapsulation ratio and the anti-tumor efficacy of liposomes [113,114]. However, low pH will induce the acid-hydrolysis of lipid (such as phosphatidylcholine), further inducing the drug leakage and stability problematic of liposomes [115].

Onivyde using a novel polyanion salt, i.e., sucrosofate triethylammonium salt (TEA-SOS), to produce the electrochemical gradient across the liposomes membrane [116,117] (Figure 4d). One molecule of polyanion salt can bind eight molecules of irinotecan. The liposomes are firstly prepared in the solution of TEA-SOS. After exchanging the extraliposomal, non-encapsulated TEA-SOS medium by the drug-loading buffer, the empty liposomes are incubated with irinotecan hydrochloride solution at a pH of 6.5 [118]. Irinotecan encapsulated in the liposome interior shows a gelated or precipitated state as a sucroseoctasulfate salt form. High encapsulation efficiency of more than 95% can be obtained.

#### 4.3.3. Drug–Lipid Conjugation by Covalently Linking

Covalently linking the drug molecules to lipids via a linker is another efficient strategy to load the drug within liposomes, e.g., Mepact [47,119]. Muramyl dipeptide (MDP) is the component of the cell wall of primarily Gram positive bacteria and shows the capability to enhance immune responses. The MDP liposome shows problems, including low entrapment efficiency and drug leakage during the storage since MDP is a water-soluble and low-molecular-weight molecule [120]. To improve the lipid solubility of MDP, MTP-PE (muramyl tripeptide-phosphatidyl ethanolamine) was synthesized by linking the MDP to PE through a peptide spacer [47,121] (Figure 4c). The amphipathic molecules of MTP-PE intercalated into the membrane bilayers of liposomes during the reconstitution of the lyophilized product (MTP-PE, POPC, and OOPS) with saline solution. MTP-PE existed within the liposomes, and no free MTP-PE was found [93].

#### 4.3.4. Combination Method

A combination of passively loading and actively loading is used for Vyxeos, which is the first approved liposome loaded with two different drugs (cytarabine and daunorubicin) in the same vesicle [122]. In brief, cytarabine is passively encapsulated into liposomes when hydrating the lipids foams with a solution of Cu(gulconate)_2_, triethanolamine (TEA), pH 7.4, and cytarabine. After sizing reduction and buffer exchange to remove the unencapsulated drug and Cu(gulconate)_2_/TEA, daunorubicin buffer solution at neutral pH is incubated with the cytarabine-loaded liposomes. The daunorubicin is actively accumulated inside liposomes using a Cu(gulconate)_2_/TEA-based loading approach. The daunorubicin diffuses through the lipid bilayer into intraliposome while the neutral form of TEA permeates towards the extraliposomal medium, establishing a kinetic and stoichiometric relationship between daunorubicin and the TEA efflux [123,124]. The Cu(gulconate)_2_/TEA plays a key role in interacting with both drugs, keeping both drug retention inside liposomes and modulating the drug release from the liposomes [36].

## 5. Critical Quality Attributions

Different with the conventional drug dosage form (e.g., injection solution for small molecules), the transport of therapeutic molecules loaded in liposomes to tumor cells after systemic administration (e.g., intravenous injection) is more complex and mainly undergo the following steps [125,126]: (a) Extravasation from intravascular space to tissue interstitium: liposomes across the discontinuous endothelial junctions (100 nm–2 µm) of tumor vascular wall via diffusion and/or convection to enter the tumor interstitium. Meanwhile, a part of liposomes are cleared from systemic circulation by MPS, especially for the particles with a large size (>200 nm), hydrophobic and charged particle surface (negative or positive charge). (b) Interstitial transport by diffusion and convection to close to the individual tumor cells. Surface modification on liposomes using active targeting will overcome the physical resistance for particle diffusion in extracellular matrix (ECM) since higher affinity are generated between targeting ligand on the particles and the receptors on the surface of tumor cells. (3) Attach to cell membrane through non-specific or specific binding. (4) Enter the cell through the endocytosis, membrane fusion or diffusion. The pathways of endocytosis is depended on the particle size, i.e., the particles with the size of 200 nm and 500 nm are clathrin- and caveolae-mediated endocytosis, and up to 5 µm for macropinocytosis. (5) Intracellular trafficking and drug release. Based on this transporting process of liposomes, Doxil pronouncedly reduces the cardiac toxicity compared to the administration of conventional doxorubicin since the circulating liposome particles are unable to cross the continuous endothelial junctions of blood vessels in the heart [127]. DaunoXome increases daunorubicin tumor delivery by about 10-fold over conventional drug and provides sustained release in vivo [21].

Critical quality attributions (CQAs) of the product are physical, chemical, biological, or microbiological properties or characteristics that would affect the product’s pharmacokinetic and pharmacodynamic performance [128]. Based on the mechanism of pharmacokinetics and the properties of liposomes, CQAs of liposomes typically include particle size and size distribution, morphology, lamellar structure, surface properties (zeta potential, PEGlated thickness, and targeting molecules, such as a ligand, if applicable), the phase transition temperature of the lipid membrane, drug loading efficiency, release rate, etc. For example, the lamellar structure of liposomes could affect the rate of drug release, and the morphology could affect the circulation time of liposomes in vivo. Here, we focus on three CQAs in this section.

### 5.1. Particle Size and Size Distribution

As mentioned above, the whole pharmacokinetic process of liposomes, such as systemic circulation and the clearance by MPS, the extravasation into tissue interstitium, interstitial transport in the extracellular matrix, and cellular uptake and intracellular trafficking, are dimension-dependent [129,130]. The particles with a size <200 nm decrease the opsonization by serum proteins and clearance of MPS. For Myocet, smaller liposomes have higher anti-tumor efficacy and increased mean survival time in a murine leukemia model [46]. Mepact with a particle size of 2.0–3.5 µm can prompt the phagocytosis by monocytes/macrophages and trigger the immunomodulatory effects for cancer therapy [92]. Singh et al. [131] found that vaccines with different particle sizes of adjuvant liposomes (the Army Liposome Formulation, ALF) resulted in different immune responses, i.e., dendritic cells more efficiently uptake small-size particles in the range of 10–200 nm, whereas other immune cells, such as macrophages, are prone to phagocytose large-size particles. Niu et al. [17] studied insulin-loaded liposomes for oral administration and found that liposomes with the diameters of 150 nm and 400 nm exhibited slower and prolonged hypoglycemic action up to 24 h, while liposomes with a particle size of about 80 nm and 2 µm exhibited a transient and no pharmacological effect, respectively.

### 5.2. Surface Modification

Liposomes coated by highly flexible PEG chains to create a hydration layer are an important tool for liposome modification (described in the Section 3.2), which reduces the clearance by MPS, prolongs the circulation lifetime, and prevent liposomes from aggregation [132]. Another common surface modification of liposomes is using ligands for active targeting (described in the Section 1).

FDA guidelines recommend that the coating thickness of nanomaterial could be described in the dossier [133] since the coverage density and thickness of the layer affect cellular uptake and control nanoparticle transport through biological matrices [134]. The reflection paper of EMA [135] mentioned that the influences of the surface coating either by a non-covalent or covalently bound on the product stability, pharmacokinetics, bio-distribution, bimolecular interaction, and receptor-mediated cellular interaction should be considered. Additionally, the coating material should be completely characterized and controlled, including its consistency and reproducibility, surface coverage heterogeneity, the orientation and conformational state of the ligand, physico-chemical stability, premature detachment, and/or degradation of the coating, etc.

### 5.3. Phase Transition Temperature

**Figure 5:**
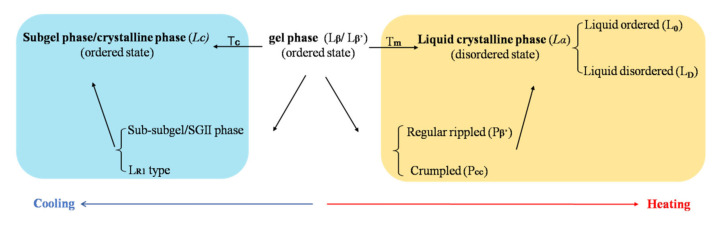
The phase transition of liposomal bilayer dispersed in aqueous solution. Heating above the melting temperature (T_m_), the phase of bilayer transits from “solid” gel phase (*L_β_*: hexagonal lattice untitled chain or *L_β’_*: quasi hexagonal array with titled chain) (ordered state) to liquid crystalline phase (*L_α_*) (disordered state). Cooling below T_c_, the phase of bilayer transits from “solid” gel phase (*L_β_* or *L_β’_*) (ordered state) to subgel phase or crystalline phase (*L_c_*) (ordered state).

The phase transition temperature of the bilayer membrane is a critical parameter for liposome production, stability during the storage, and drug release in vivo. A large number of investigations on the phase transition have been completed [65,136,137,138]. Hydrated lipid bilayers exhibit three lamellar forms: a crystal phase (*L_C_*), a “solid” gel phase (*L_β_*: hexagonal lattice untitled chain or *L_β_’*: quasi hexagonal array with titled chain), and a liquid-crystal phase (*L_α_*) [139], shown in Figure 5. In the lamellar gel phase, the acyl chains are preferentially aligned in an all-trans conformation, and lateral diffusion is very slow. Cooling under the transition temperature of Tc, the lamellar changes from a gel phase to *L_C_* phase. *L_C_* is also called the subgel phase; the hydrocarbon chains are in a fully extended, all-trans conformation, and the polar head groups are relatively immobile. Between the transition from the gel phase to *L_C_*, the metastable precursor SGII phase (also known as sub-subgel) or L_R1_ phase might occur [140]. Heating the temperature over the T_m_ (melting transition temperature), the membrane changes from the order state (gel state) to a relatively disordered state (*L_α_*), and the hydrocarbon chains show rapid trans-gauche fluctuations, leading to an increase in the permeability of membrane and drug molecules cross the membrane easily.

Normally, a higher T_m_ of lamellar than physiological temperature (37 °C) is required. Thus, the rate of drug molecules crossing the gel state of the membrane remains slow. The burst release and drug leakage from liposomes in vivo can be better prevented in order to reduce the risk of systemic toxicity.

## 6. Regulatory Consideration

Over the last few decades, approximately 100 nanomedicines and 11 nanomedicines have been approved by the FDA and EMA, respectively, while 48 nanomedicines are presently under clinical trials in the European Union [109]. Considering the increasing number of nanomedicine applications and sharing the experience of the regulatory network in the scientific evaluation of nanomedicines, several guidelines about nanomaterial and nanoproducts were released by the FDA, EMA, Ministry of Health, Labour and Welfare—Japan (MHLW), and Chinese National Medical Products Administration (NMPA). These guidelines involve different nano-dosage forms, including liposomes, iron-based nano-colloidal products, block copolymer micelles, and nucleic-acid (siRNA)-loaded nanoproducts (Table 3). Among these guidelines, all four regulatory agencies worked out the guidance about liposomes, which might be attributed to the relatively common dosage form and the relatively large numbers approved in market and clinical trials.

Given the structure’s complexity and great diversity in liposomal products, the ultimate goal of quality, safety, and effectiveness should be kept in mind in each stage of the product’s life cycle. Based on these guidelines, we emphasize that building a comprehensive knowledgebase to better understand potential risk generated during manufacturing, analysis, and material control on the physicochemical and biological characteristics of a product is extremely important. Knowledge can be gained from the early stages of pharmaceutical research and development, can also be updated from subsequent manufacturing and associated control strategy over time. The deeper understanding of the relationships among critical material attribution (CMA), critical process parameters (CPP), physicochemical properties, and in vivo performance of liposomes, the lower risk yields [128]. Secondly, excipient, especially for lipids, plays an important role in the quality of liposomal products. A minor change of lipid materials might induce the variation of pharmacokinetic or pharmacodynamics of the drug, potentially leading to serious toxicity. The requirements of lipid control, including lipid source (extraction or synthesis), characteristic, specification, and stability, are described in detail in FDA guidance. Thirdly, sterilization is considered a challenging process for liposome manufacturing since most liposomal products are intended for parenteral administration. Sterile filtration using 0.22 µm membrane is commonly adopted in the pharmaceutical industry. However, issues such as membrane clogging, reduced integrity of liposomes, as well as ineffective retention of small bacteria may occur [141]. Therefore, the promising sterilization method and the validation of the sterilization process are critical for batch consistency as well as sterility assurance of liposome products.

In addition, the in vivo fate of liposomal carriers is another critical consideration for the development of liposome preparations because the leakage of the payload from nanoparticles may be even quicker than we have recognized [142]. A guideline for non-clinical pharmacokinetics of nanomedicine recently released by the Center for Drug Evaluation (CDE), NMPA, encourages in vivo measurement of vehicles besides cargos [143]. Fluorescent labeling is the most commonly used technique to monitor the in vivo transport of vehicles. However, it is crucial to discriminate intact vehicles from free fluorescent molecules that are released from nanoparticles [144]. Aggregation-caused quenching (ACQ)isa promising method to eliminate free-probe interference due to the environment-responsiveness characteristics, although the phenomenon was generally regarded to be unfavorable in bioimaging [145].The ACQ probes emit near-infrared fluorescence when they are loaded in carrier matrix (molecularly dispersed in general), but quench immediately and absolutely once they are released into the aqueous environment due to π-πstacking. Therefore, the fluorescence indicates the intact vehicles. The in vivo fates of various nanoparticles (e.g., polymeric nanoparticles, micelles, nanoemulsions, and nanocrystals) via different routes (e.g., oral, intravenous, transdermal, nasal, and ocular routes) have been explored by using the ACQ probes [146,147,148,149].

## 7. Future Perspectives and Concluding Remarks

**Figure 6:**
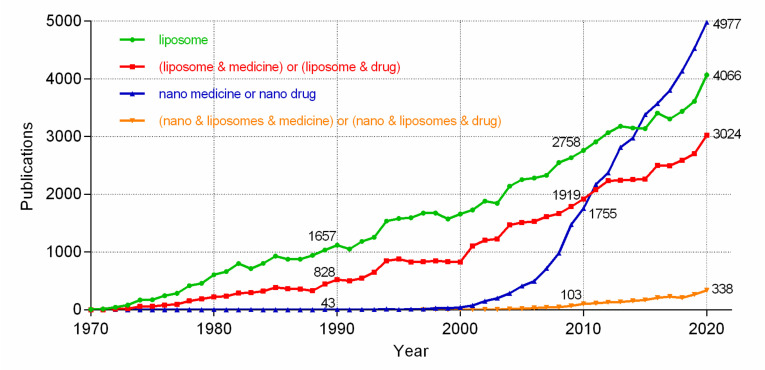
The comparison profiles of publications using setting TITLE-ABS-KEY as “liposome”, “(liposome AND medicine) or (liposome AND drug)”, “(nano AND liposomes AND medicine) or (nano AND liposomes AND drug) and “(nano AND medicine) or (nano AND drug)” in the year range between 1970 and 2020 in Scopus.

We compared the number of publications setting TITLE-ABS-KEY as “liposome”, “(liposome AND medicine) or (liposome AND drug)”, “(nano AND liposomes AND medicine) or (nano AND liposomes AND drug), and “(nano AND medicine) or (nano AND drug)” in the year range between 1970 and 2020 in Scopus. Interesting results were found. From Figure 6, we can conclude that (1) liposome as a drug carrier and applied in other fields (e.g., food, cosmetics) starts earlier than nanomedicines, i.e., the year 1970 vs. 1990. (2) The application of liposomes as medicine carriers in total liposomal publications increases over time, i.e., 50% in 2000, 70% in 2010, and 74% in 2020. (3) Although the development of nanomedicine started later than the use of liposomes, the number of publications about nanomedicine shows an exponential increase over time. (4) Extremely low percentages (7%) of medicine/drug nanoliposomes in total nanomedicine/drug are observed, which might be false data, since there are 3024 publications about liposome medicine in the year 2020. We speculate that the name combination of “nano” and “liposome” might be less frequently used compared with the names of nanoparticles, nanocrystals, or nanosuspensions. It was reported by the FDA that more than 500 liposome applications were received up until 18 February 2016 [79]. Among these applications, except for around 100 submissions applied for combination therapy of liposome with another therapeutic, the remaining submissions (used individually) were 3% NDAs, 1% ANDAs, and 96% INDs. The data collected from the laboratory level and pharmaceutical industry indicate that there will be a large number of liposomal products transformed from the laboratory bench to pilot plant and market in the near future.

From the first liposome product of Doxil approved in 1995, liposome techniques have been further developed for more than 20 years. We summarize the successful experience and pain points based on the abundance of publications and commercial products. Liposomes can be well-designed and display intended functions depending on human requirements and needs. On the one hand, there are major obstacles during the development and commercialized production, such as the individual differences in the EPR effect, accelerated blood clearance (ABC) phenomenon of PEGylated liposomes, scale-up, the reproducibility/consistency among different batches and manufacturing sites, and excipient control.

On the other hand, a large number of smart liposomal systems are developing in laboratory or undergoing clinical trials, such as active targeting liposomes (e.g., anti-EGFR immunoliposomes, phase II; MBP-426, phase II) and stimuli-sensitive liposomes (e.g., ThermoDox) [150]. The microenvironment at the target disease site can be exploited to trigger the release of drug from liposome carrier. The external or internal stimuli such as temperature, pH, light, eletromagnetic fields, enzyme, and hypoxia, are frequently studied as “on-off” switch of the drug release [151,152]. Although promising results were obtained in pre-clinical studies, it is challenging for successful clinical translation due to the major issues, such as the leakage of the cargo before reaching the target sites, the individual differences among patients, as well as the multi-modal therapies involved. ThermoDox, the fastest developed thermosensitive liposomes, got a failure at the second Phase III clinical trial, designed for combination with radiofrequency ablation for the treatment of hepatocellular carcinoma [153]. However, the failure just means the failure of liposomal product under the certain clinical design, and these smart techniques will present many new opportunities for liposomes to further increase the therapeutic efficacy and decrease the side-effects.

A topic about “where are we in the development path of nanomedicines” has been widely argued recently. When looking back through history on the application situation and the performance of liposomes, we maintain a positive attitude. Three types of liposomal products have been approved by the Chinese NMPA, i.e., Lipusu (paclitaxel liposome), doxorubicin hydrochloride liposome, and Amphotericin Bliposome. Additionally, both large pharmaceutical industries and small innovative companies in China are developing nanomedicines, including liposomes, nanocrystal, inorganic particles, and polymeric micelles. At the same time, the topic of the “safety and quality evaluation of nanomedicine” has been selected as a key project for developing the regulatory science by NMPA in 2019. We are preparing a regulatory framework to respond to the future of nanomedicines with a seamless connection.

## Figures and Tables

**Table 1:** Summary of liposomal products approved by FDA and EMA.

Product Name	API	Approved Year/Area	Dosage Form	Adm. Route	Indication
Doxil Caelyx	Doxorubicin hydrochloride (DOX·HCl)	1995, US 1996, EU	Suspension	IV	Ovarian cancer, Kaposi’s sarcoma, myeloid melanoma
DaunoXome	Daunorubicin	1996, US	Suspension	IV	Kaposi’s sarcoma
AmBisome	Amphotericin B (AmpB)	1997, US	Lyo	IV	Systemic fungal infection
DepoCyt DepoCyte	Cytarabine	1999, US 2001, EU	Suspension	IT	Lymphomatous meningitis
Myocet	DOX·HCl	2000, EU	3 vials	IV	Breast cancer
Visudyne	Verteporfin	2000, US 2000, EU	Lyo	IV	Wet AMD
DepoDur	Morphine	2004, US	Suspension	Epidural	Postoperative pain
Mepact	MTP-PE	2009, EU	Lyo	IV	Osteosarcoma
Exparel	Bupivacaine	2011, US 2020, EU	Suspension	Local infiltration	Post-surgical analgesia
Marqibo	Vincristine Sulfate	2012, US	3 vials	IV	Leukemia
Onivyde	Irinotecan hydrochloride trihydrate	2015, US 2016, EU	Suspension	IV	Pancreatic adenocarcinoma
Vyxeos	Daunorubicin, cytarabine	2017, US 2018, EU	Lyo	IV	Leukemia
Shingrix	Recombinant varicella-zoster virus glycoprotein E	2018, EU	2 vials (powder and suspension)	IM	Against shingles and post-herpetic neuralgia
Arikayce	Amikacin sulfate	2018, US 2020, EU	Suspension	Oral inhalation	Lung disease

This list is only for liposomal forms approved by FDA and EMA, excludes generics (e.g., doxorubicin hydrochloride (liposomal), lipid complexes (e.g., Abelcet, Amphotec, and Onpattro), and also excludes the nationally authorized liposomal products in Europe. Abbreviations: intravenous infusion (IV), intramuscular injection (IM), intrathecal injection (IT), lyophylization (Lyo), muramyl tripeptide phosphatidyl ethanolamine (MTP-PE).

**Table 2:** The particle size, structure, and lipid components of the commercial liposomal products.

Product Name	Size	Structure	Main Composition
Doxil/Caelyx	ca. 100 nm	SUVs	HSPC, MPEG-DSPE, Chol
AmBisome	50–100 nm [41]	SUVs	HSPC, DSPG, Chol
DaunoXome	45–80 nm [42]	SUVs	DSPC, Chol
Marqibo	130–150 nm	SUVs	SM, Chol
Onivyde	ca. 110 nm	SUVs	DSPC, MPEG2000-DSPE, Chol
Visudyne	<100 nm	SUVs [43]	EPC, DMPC
Shingrix	50–100 nm [44]	SUVs	DOPC, Chol
Arikaye	200–300 nm [45]	LUVs	DPPC, Chol
Vyxeos	ca. 110 nm	bilammer	DSPC, DSPG, Chol
Myocet	80–90 nm [42]	MLVs [46]	EPC, Chol
Mepact	2.0–3.5 μm [47]	MLVs	POPC, OOPS
DepoCyt	20 μm [42]	MVLs	DOPC, DPPG, Chol, triolein
Exparel	24–31 μm [42]	MVLs	DEPC, DPPG, Chol, tricaprylin
DepoDur	17 to 23 μm	MVLs	DOPC, DPPG, Chol, triolein, tricaprylin

Abbreviations: fully hydrogenated soy phosphatidylcholine (HSPC), egg phosphatidylcholine (EPC), distearoylphosphatidylcholine (DSPC), dioleoyl phosphatidylcholine (DOPC), dierucoyl phosphatidylcholine (DEPC), palmitoyl-2-oleoyl-sn-glycero-3-phosphocholine (POPC), dipalmitoyl phosphatidylcholine (DPPC), dimyristoylphosphatidylcholine (DMPC), dipalmitoylphosphatidylglycerol (DPPG), distearoylphosphatidylglycerol (DSPG), dioleoyl phosphatidylserine(DOPS), dioleoylphosphatidylserine (OOPS), cholesterol (Chol), sphingomyelin (SM), N-(carbonyl-methoxypolyethlyeneglycol-2000)-distearolyphosphatidylethanolamine (MPEG-2000-DSPE).

**Table 3:** Nanomedicine guidelines published by FDA, EMA, MHLW, and NMPA.

Regulatory Agency	Nanomedicine Guidelines
FDA	Guidance for industry: Considering whether an FDA-regulated product involves the application of nanotechnology; Drug products, including biological products, that contain nanomaterials guidance for industry (draft); Guidance for industry: liposome drug products chemistry, manufacturing, and controls; human pharmacokinetics and bioavailability; and labeling documentation.
EMA	Data requirements for intravenous iron-based nano-colloidal products developed with reference to an innovator medicinal product; Surface coatings: general issues for consideration regarding parenteral administration of coated nanomedicine products; Data requirements for intravenous liposomal products developed with reference to an innovator liposomal product; Development of block-copolymer-micelle medicinal products—Joint EMA and MHLW; Non-clinical studies for generic nanoparticle iron medicinal product applications.
MHLW	Development of block-copolymer-micelle medicinal products—Joint EMA and MHLW; Guideline for the Development of Liposome Drug Products; Reflection paper on nucleic acids (siRNA)-loaded nanotechnology-based drug products.
NMPA	Guideline for the quality control of nanomedicines (draft); Guideline for the non-clinical pharmacokinetics of nanomedicines (draft); Guideline for the non-clinical safety evaluation of nanomedicines (draft).
